# Experiments and Observations, &c.

**Published:** 1781-08

**Authors:** 


III. *Experiments and Obfervations*, &c. *By* Jofeph Prieftley, L. L. D. F. R. S. Vol. II. *(Continued from page 9.)*

IN the 6th and 7th fections Dr. Prieftley treats of the air produced by fubftances putrefying in water and in quickfilver. The experiments in this and the following fection were entered upon chiefly to difcover the *principle of nutrition* in vegetable and animal fubftances, and they feem to lead us to fuppofe that this principle is phlogifton in fuch a ftate as to be capable of becoming, by putrefaction, a true inflammable air. The author finds that fubftances putrefying in water yield many times more air than when they putrefy in quickfilver.

In the 8th fection the author fhews that iron filings and brimftone, made into a pafte with water, water, yield inflammable air; and he is led to ſuſpect, that air is never phlogiſticated but by means of inflammable air, either in its *naſcent*, or *perfect* ſtate.

Dr. Ingenhouſz, Signor Moſcati, and Mr. Cruikſhank affirm, that *perſpination* as well as *reſpiration* injures the ſurrounding air, converting it either into fixed or phlogiſticated air: but Dr. Prieſtley in the 9th ſection ſhews, by more careful and accurate experiments, that theſe gentlemen were miſtaken. In the 10th ſection he refutes the opinion that the fixed air, diſcovered by reſpiration, is a compound of common air and phlogiſton, and ſhews that it is only precipitated from the common air. It has uſually been believed, that a man phlogiſticates or *conſumes* a gallon of air in a minute; but Dr. Prieſtley reduces it to a fourth part of that quantity.

In the 11th ſection he treats of the fixed air diſcovered by putrefaction. From the experiments related it ſeems to appear, that the whole of it is precipitated from the common air by the inflammable principle; that it is not leſs than *a fifth* part of the whole; and of courſe that the *whole diminution* is owing to this precipitation of fixed air. He relates ſome inſtances however where the diminution was completed at once, and and yet no fixed air was found; and acknowledges himself at a loss to account for so singular a phenomenon. *Quere*, Whether it did not unite with the *alkaline* basis of the inflammable air into a minute quantity of fixed substance, (solid or fluid) in the same manner as marine acid and alkaline air unite and condense into common sal ammoniac?

The 12th section treats of several unexpected changes, produced in various kinds of air, by the same processes. And the experiments related in the 13th confirm the author's former observation, that fishes phlogisticate the common or dephlogisticated air contained in water.

The 14th section contains some new experiments concerning the production and constitution of dephlogisticated air. Dr. Priestley observes, that it is obtained with the least trouble and expence from nitre; and from the experiments adduced it appears, that it is composed of a principle common to the nitrous and vitriolic acids, and about a twelfth part of an earth. By experiments described in the close of this section it appears, that mercury is more easily reduced to a calx, or precipitate *per se*, in proportion as the air which surrounds it is free from phlogiston.

At

At the end of Dr. Ingenhousz's late treatise, an important discovery of the Abbé Fontana's is annexed; and it is asserted as a *fact*, that by breathing dephlogisticated air through lime water it will serve for respiration thirty times as long as in the ordinary way, by reason that the lime water frees it from *fixed air*. The experiments related in the 15th section demonstrate that both the *fact* and the *reasoning* urged in support of it are void of foundation.

The 16th section contains several observations relating to fixed air; and the 17th treats of the state of air in water. In the 18th are given some new observations on nitrous air; and in the 19th the author considers the phenomena of the mixture of nitrous and common air, with a view to the improvement of eudiometers. He had formerly observed, that if after having mixed equal quantities of the two kinds of air in a wide jar, he made them ascend very quickly into the long tube on which the measures were marked, the diminution was more considerable than when he made them ascend more slowly. This difference he since finds to arise from part of the undecompoſed nitrous air being absorbed by the water in the latter case. Dr. Ingenhousz had published an account of the Abbé Fontana's

 eudioeudiometrical method, by which it was pretended that the ſtrength of the nitrous air was a matter of no conſequence, and which therefore would have been a very advantageous diſcovery: but Dr. Prieſtley has ſhewn this to have been an error.

In the 20th and 21ſt ſections the author treats of a ſpecies of air which he had formerly diſcourſed of, in which a candle burns with an enlarged flame, and ſometimes alſo with a crackling noiſe, and which therefore he now calls *dephlogiſticated nitrous air.* This air he thinks conſiſts of a dephlogiſticated nitrous vapour diffuſed through a quantity either of nitrous or phlogiſticated air; for the former may be ſeparated by means of water, the latter remaining behind. This air, though it ſerves for combuſtion, is fatal to animal life; which the author ingeniouſly accounts for, from the known facts that many ſubſtances can act on one another when they are hot, which do not at all affect each other when cold. This air may be obtained in large quantities, by precipitating a ſolution of copper in ſpirit of nitre with iron: and alſo by other methods.

In the 22d ſection ſome curious experiments are related, by which it appears that alkaline air is is capable of being converted into genuine, inflammable air by means of the electric spark. The author infers from hence, that the basis of inflammable air is not an acid, as he once imagined, but an alkali. Mr. Bewley in his ingenious observations on several parts of this volume, inserted in the appendix, renders it probable that the electric spark in this case acts only by its *heat*.

In the 23d section the author gives some new instances of the great volatility of mercury, which is known to evaporate *in vacuo*, in the common temperature of the atmosphere. Mr. Bewley in his remarks on this section at the end of the volume has also given his own observations on this curious subject. The 24th section contains a variety of experiments made with the nitrous acid and metallic calces.

The 25th section treats of the mixture of nitrous and vitriolic acid. One of the most extraordinary circumstances attending this mixture is the extreme volatility that it gives to the nitrous acid, the whole of it making its escape. This separation may also be effected by expressing the mixture to nitrous air, which phlogisticates the nitrous acid, and thereby renders it extremely volatile. The marine acid, when mixed with vitriolic acid, alſo eſcapes in the form of air.

The 26th ſection treats of the marine acid, and marine acid air. The author finds that if this acid be dephlogiſticated, it will not aſſume the form of air; and of courſe that a certain quantity of phlogiſton is neceſſary to form the marine, as well as the nitrous and vitriolic acids, into airs.

The 27th ſection is the author's paper on the *lateral electric exploſion*, which was printed in the 60th volume of the Philoſophical Tranſactions. The 28th contains ſome new experiments in electricity, particularly on the ſuppoſed non-conducting power of water and quickſilver when in the ſtate of vapour.

In the 29th ſection the author relates ſome experiments which he had made on ſound in different kinds of air: the reſult of which was, that the intenſity of ſound depends ſolely on the *denſity* of the air in which it is made, without any regard to its chemical properties. The vapour of water is alſo known to be a medium of ſound.

The 30th ſection contains miſcellaneous experiments; and, amongſt others, that air rendered exceedingly offenſive to the noſtrils by putreputrefaction is not always properly phlogisticated; the particles which affect the smell not being combined with, but only diffused through the air, and may be separated by passing the air through water.

The 31st section consists of remarks on the preceding volumes of the author's *Observations on Air*, explaining and correcting them by the help of subsequent experiments and observations. And the 32d section contains a summary of all the most remarkable facts in the present and four preceding volumes, digested under proper heads, with references to the volumes and pages; which those who have occasion to consult these volumes will find exceedingly useful.

The 33d and last section contains experiments made after the preceding sections were sent to press; and, amongst others, on the power of *conducting heat* in different kinds of air. The author found that inflammable air conducted heat much better than any other kind; fixed and acid air considerably worse than common air; alkaline air rather better than acid; and dephlogisticated air a little worse than common air. In making these experiments he observed, that alkaline air was remarkably expanded by the heat, agreeably to his former observations on that subject.

An

An Appendix is subjoined to the volume containing letters on philosophical subjects from several of the author's friends. The first of these is from Mr. Arden, giving an account of his having imitated the phenomenon of the *fire ball* by artificial electricity. The 2d is from Mr. Bewley, containing observations on several parts of the volume, together with experiments on the fixed air, or mephitic acid discovered by respiration. The 3d is from Mr. Watt, and also contains observations on different parts of this volume. The 4th is from Dr. Withering, describing a new apparatus for impregnating water with fixed air. The 5th is from Mr. Warltire on the firing of inflammable air in *close vessels*. He finds that there is a *loss of weight* in these cases, and this he attributes to the latent heat, or fire, which is extricated and dispersed in the operation. If this be true, we are furnished with a proof that fire is actually heavy: the fact however requires to be confirmed by repeated and diversified trials; and we heartily recommend it to Mr. Warltire to prosecute so extraordinary an experiment.

We have thus given as ample an abstract of this excellent work as the nature of our plan will permit; but for an account of the author's numerous numerous experiments, and a variety of other interefting particulars which are not here noticed, we are obliged to refer our readers to the performance itfelf.

